# Impact of a Public Health Policy on Accessibility to Levodopa for People with Parkinson's Disease in Brazil

**DOI:** 10.1002/mdc3.70494

**Published:** 2026-01-06

**Authors:** Juliana dos Santos Duarte, Laís Resque Russo Pedrosa, Rafael Sidônio Gibson Gomes, Bruno Lopes Santos‐Lobato

**Affiliations:** ^1^ Laboratório de Neuropatologia Experimental Universidade Federal do Pará Belém Brazil

**Keywords:** Brazil, health policy, levodopa, Parkinson's disease

Parkinson's disease (PD) is the second most common neurodegenerative disease in the world and the fastest‐growing neurological disorder.^1^ Yet in many low‐ and middle‐income countries, levodopa remains inconsistently available and often unaffordable.^2^ In Brazil, the Popular Pharmacy Program (PPP) was created in 2004 and dispenses essential medicines through accredited private pharmacies across the country. Levodopa (immediate‐release) has been included since 2012 under co‐payment, and in 2024 became fully subsidized (zero co‐pay) (Supporting Information).

We evaluated the nationwide impact of the PPP on the accessibility to levodopa through the state‐level data on dispensed tablets of immediate‐release levodopa/benserazide (L/B, 25/100 mg) and carbidopa/levodopa (L/C, 25/250 mg) in Brazil from 2020 to 2024. Also, we mapped the distribution of PPP units per municipality and calculated the patient‐equivalent estimates as a medication proxy for the number of people with PD based on average daily levodopa use.^3^ Methods are described in the Supporting Information.

Overall, L/B dispensing increased 25.94% from 2020 to 2024 (annual rate 5.93%), while L/C decreased 12.9% (annual rate—4.5%) (Fig. 1). In 2024, PPP levodopa dispensing corresponded to 73,576 L/B and 1612 L/C patient‐equivalents (Tables S1 and S2), about 15% of an estimated 500,000 Brazilians living with PD^4^, at a public cost on the order of US$29 million per year (approximately US$400 per patient‐equivalent per year) (Supporting Information). The PPP units were unevenly distributed: South/Southeast regions concentrated more units, whereas North/Northeast had fewer (Table S3). Many municipalities—36.44% in the North region—had no PPP units (Fig. S1). These patterns suggest that the PPP is consistent with increased access to levodopa but also presents substantial geographic inequities.

**Figure 1:**
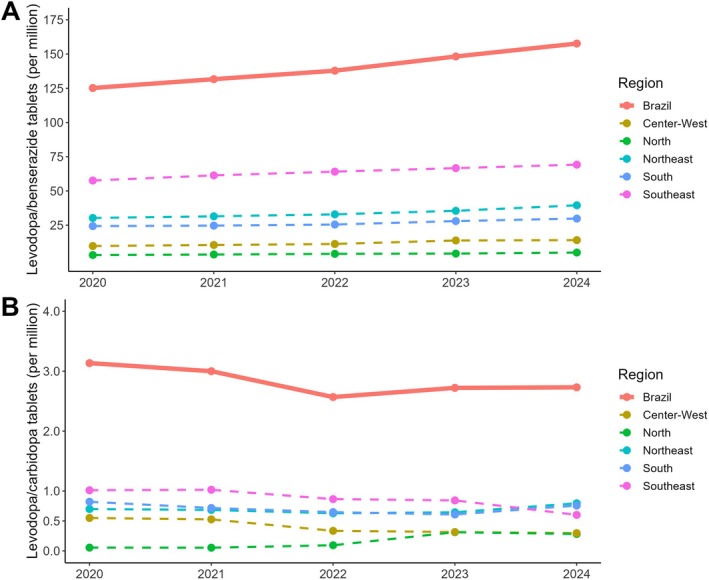
Number of levodopa tablets dispensed from 2020 to 2024 by region through the Brazilian Popular Pharmacy Program. (A) Number of benserazide hydrochloride 25 mg + levodopa 100 mg tablets. (B) Number of carbidopa 25 mg + levodopa 250 mg tablets. The number of tablets is represented in millions of units.

To the best of our knowledge, this is the first study to analyze the nationwide impact of a public health policy on the accessibility to levodopa. As limitations, we did not have access to levodopa dispensing data at the municipal level. Furthermore, some municipalities occasionally dispense levodopa formulations free of charge, but these numbers could not be quantified. The lack of data on the number of people with PD by state also limits the ability to adjust estimates of levodopa tablet distribution according to the affected population. Similarly, the absence of regional data on hospitalization and mortality rates among people with PD impairs the assessment of policy impact on health indicators. Also, we could not evaluate the proportion of levodopa dispensed for other conditions, such as atypical parkinsonisms.

In conclusion, the Brazilian PPP is associated with a greater accessibility to levodopa, despite the limited number of individuals benefiting from the health policy and regional inequalities. Among measures for improving access to levodopa, public health managers should prioritize ensuring that all municipalities are covered by at least one PPP unit and promoting awareness of the policy for people with PD and healthcare providers. These results may guide other low‐ and middle‐income countries to elaborate new health policies aiming to increase accessibility to levodopa for their people with PD.

## Author Roles

(1) Research project: A. Conception, B. Organization, C. Execution; (2) Statistical Analysis: A. Design, B. Execution, C. Review and Critique; (3) Manuscript: A. Writing of the first draft, B. Review and Critique.

J.S.D.: 1A, 1B, 1C, 2A, 2C, 3B

L.R.R.P.: 1C, 2B, 3B

R.S.G.G.: 1C, 2B, 3B

B.L.S‐L.: 1A, 1B, 1C, 2A, 2B, 2C, 3A, 3B

## Disclosures

**Ethical Compliance Statement:** The authors confirm that the approval of an institutional review board was not required for this work. For this work, obtaining informed consent was not required. We confirm that we have read the Journal's position on issues involved in ethical publication and affirm that this work is consistent with those guidelines.

**Funding Sources and Conflict of Interest:** No specific funding was received for this work. The authors declare that there are no conflicts of interest relevant to this work.

**Financial Disclosures for the Previous 12 Months:** JSD reports a research grant funded by the Brazilian Federal Agency for Support and Evaluation of Graduate Education. BLS‐L reports a research grant funded by the Brazilian National Council for Scientific and Technological Development.

## Supporting information


**Data S1. Supporting Information content** The Supplementary materials contain (I) Regulatory framework related to the Brazilian Popular Pharmacy Program and (II) Methods.

**Supplementary Figure S1.** Map of the distribution of private pharmacies accredited by the Brazilian Popular Pharmacy Program per municipality in 2025. Areas in darker red indicate municipalities with a higher concentration of units, while white areas represent municipalities with no registered units.

**Supplementary Table S1.** Distribution of the number of levodopa tablets through the Brazilian Popular Pharmacy Program from 2020 to 2024, by state and region.

**Supplementary Table S2.** Distribution of the patient‐equivalent estimates of people with Parkinson's disease receiving levodopa through the Brazilian Popular Pharmacy Program from 2020 to 2024, by state and region (including sensitivity analyses).

**Supplementary Table S3.** Number of Popular Pharmacies units, population over 50 years, and municipalities without Popular Pharmacies in Brazil, by region and state.

## Data Availability

The data that support the findings of this study are available from the corresponding author upon reasonable request.
