# Supplementary material for: Impact of a Public Health Policy on Accessibility to Levodopa for People with Parkinson's Disease in Brazil
Source: Mov Disord Clin Pract. 2026 Jan 6;13(5):1356–8. doi: 10.1002/mdc3.70494 (PMC13172773; doi:10.1002/mdc3.70494)
Supplement: Supplementary file 1 — Data S1. Supporting Information content The Supplementary materials contain (I) Regulatory framework related to the Brazilian Popular Pharmacy Program and (II) Methods. [file MDC3-13-1356-s003.docx]

**Supplementary materials**

**Regulatory framework related to the Brazilian Popular Pharmacy Program**

1. Brazilian Ministry of Health. Ordinance No. 971, May 15, 2012: Regulates the Brazilian Popular Pharmacy Program [Website; Portuguese]; 2012. <https://bvsms.saude.gov.br/bvs/saudelegis/gm/2012/prt0971_15_05_2012.html>.

2. Brazilian Ministry of Health. GM/MS Ordinance No. 4,811, July 4, 2024 [Website; Portuguese]; 2024. <https://bvsms.saude.gov.br/bvs/saudelegis/gm/2024/prt4811_04_07_2024.html>.

3. Brazilian Ministry of Health. Consolidation Ordinance No. 5, September 28, 2017: Consolidation of rules on health actions and services of the Unified Health System [Website; Portuguese]. <https://www.gov.br/saude/pt-br/assuntos/saude-de-a-a-z/s/saude-da-pessoa-com-deficiencia/legislacao/portaria-de-consolidacao-no-05-de-28-de-setembro-de-2017.pdf>.

**Supplementary Methods**

This is a nationwide ecological study of the geographic distribution of Popular Pharmacy Program (PPP) units and levodopa dispensing in Brazil. To assess the geographic distribution of PPP units, the complete addresses of each PPP unit accredited by the PPP were obtained from the official website of the Federal Government in July 2025.^1^ The number of PPP units was then quantified by municipality and state, and secondary data tables were imported into QGIS software (version 3.28 Firenze) to map PPP coverage across the national territory. We extracted data on the population aged over 50 years from the 2022 Demographic Census.^2^

For the analysis of levodopa dispensing by the Popular Pharmacy Program, data were collected from the Brazilian Ministry of Health under the Access to Information Law, covering the period from 2020 to 2024. The tables provided by the Ministry of Health contained the number of pills dispensed per state for each reference year. The medications included in the analysis were the immediate-release levodopa/benserazide (L/B, 25/100 mg) and carbidopa/levodopa (L/C, 25/250 mg). Levodopa is rarely prescribed for conditions other than PD. Thus, we assumed that dispensed tablets primarily served individuals with Parkinson’s disease (PD).

The primary outcome was the annual number of dispensed tablets of L/B and L/C by state (2020–2024). We defined the annual number of PPP units by state and the patient-equivalent estimates of people receiving L/B and L/C in 2024 as secondary outcomes. We calculated absolute change, percent change, and annual growth rate over the period.

We derived patient-equivalent estimates (as a medication proxy for the number of people with PD) by dividing the dispensed number tablets of L/B and L/C per year by the average daily tablet use in Brazil from Bovolenta et al. (L/B: 5.83 tablets/day, equivalent to 2,143 tablets/year; L/C: 4.53 tablets/day, equivalent to 1,694 tablets/year).^3^ We performed sensitivity analyses varying the assumed daily tablet use by ±10%.

A former report from the Brazilian Federal Court of Accounts indicated that 6.57% of the total budget of the PPP for 2021 (US$443,712,747, according to the exchange rate from December 2021) was for antiparkinsonian drugs.^4^ Thus, we calculated the order-of-magnitude cost of the PPP for the Brazilian government by multiplying the total budget of PPP in 2021 by 6,57%. And considering the patient-equivalent estimates for L/B and L/C, we calculated the cost of the public health policy per patient-equivalent per year.

Statistical analyses were conducted using SPSS for Windows version 23.0 (SPSS Inc., Chicago, USA) and R (4.0.4) with the *ggplot2* package.

**References**

1. Brazilian Ministry of Health. Accredited Pharmacies under the Brazilian Popular Pharmacy Program [Website; Portuguese]; 2025. <https://www.gov.br/saude/pt-br/composicao/sectics/farmacia-popular/arquivos/farmacias_credenciadas_pfpb_atualizada.xlsx/view>.

2. Brazilian Institute of Geography and Statistics (IBGE). 2022 Demographic Census [Website]; 2025. <https://www.ibge.gov.br/cidades-e-estados>.

3. Bovolenta TM, Schumacher-Schuh AF, Santos-Lobato BL, et al. Average annual cost of Parkinson’s disease in a Brazilian multiethnic population. Parkinsonism Relat Disord 2023; 117:105897.

4. Brazilian Federal Court of Accounts. Audit Report – Assessment of the Brazilian Popular Pharmacy Program, 2022 [Website; Portuguese]; 2022. <https://portal.tcu.gov.br/data/files/36/A1/6F/F7/90F96810ED256058E18818A8/002.450-2022-4-VR%20-%20MON_Farmacia_popular.pdf>.
