# Supplementary figures and images for: Impact of a Public Health Policy on Accessibility to Levodopa for People with Parkinson's Disease in Brazil
Source: Mov Disord Clin Pract. 2026 Jan 6;13(5):1356–8. doi: 10.1002/mdc3.70494 (PMC13172773; doi:10.1002/mdc3.70494)

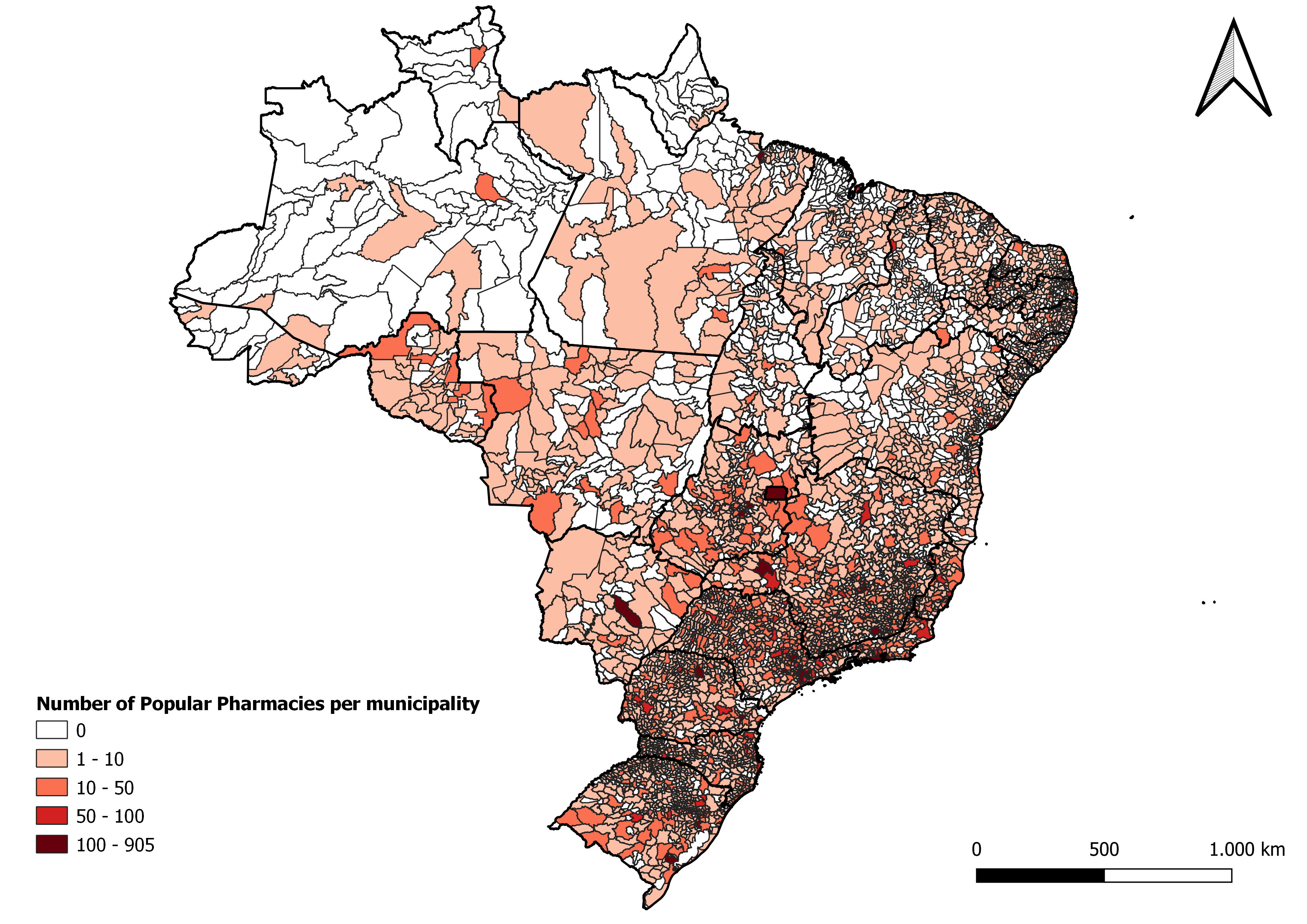

Supplement: Supplementary file 2 — Supplementary Figure S1. Map of the distribution of private pharmacies accredited by the Brazilian Popular Pharmacy Program per municipality in 2025. Areas in darker red indicate municipalities with a higher concentration of units, while white areas represent municipalities with no registered units. [file MDC3-13-1356-s004.tiff]
