# Supplementary material for: Impact of a Public Health Policy on Accessibility to Levodopa for People with Parkinson's Disease in Brazil
Source: Mov Disord Clin Pract. 2026 Jan 6;13(5):1356–8. doi: 10.1002/mdc3.70494 (PMC13172773; doi:10.1002/mdc3.70494)
Supplement: Supplementary file 4 — Supplementary Table S2. Distribution of the patient‐equivalent estimates of people with Parkinson's disease receiving levodopa through the Brazilian Popular Pharmacy Program from 2020 to 2024, by state and region (including sensitivity analyses). [file MDC3-13-1356-s001.docx]

**Supplementary Table 2.** Distribution of the patient-equivalent estimates of people with Parkinson’s disease receiving levodopa through the Brazilian Popular Pharmacy Program from 2020 to 2024, by state and region (including sensitivity analyses).

**A. Patient-equivalent estimates of people receiving L/B (5.83 tablets/day) and L/C (4.53 tablets/day) from 2020 to 2024:**

| **State** | **eL/B 2020** | **eL/B 2021** | **eL/B 2022** | **eL/B 2023** | **eL/B 2024** | **eL/C 2020** | **eL/C 2021** | **eL/C 2022** | **eL/C 2023** | **eL/C 2024** |
| --- | --- | --- | --- | --- | --- | --- | --- | --- | --- | --- |
| **Southeast Region** | | | | | | | | | | |
| Espírito Santo | 1,014 | 1,113 | 1,147 | 1,247 | 1,180 | 22 | 43 | 81 | 151 | 27 |
| Minas Gerais | 6,240 | 6,520 | 6,961 | 7,093 | 7,212 | 186 | 187 | 148 | 131 | 133 |
| Rio de Janeiro | 8,362 | 9,131 | 9,188 | 9,755 | 10,066 | 145 | 134 | 85 | 70 | 55 |
| São Paulo | 11,267 | 11,879 | 12,619 | 13,014 | 13,840 | 243 | 237 | 194 | 143 | 137 |
| **Subtotal** | **26,883** | **28,643** | **29,915** | **31,109** | **32,298** | **596** | **601** | **508** | **495** | **352** |
| **South Region** | | | | | | | | | | |
| Paraná | 2,866 | 2,923 | 3,008 | 3,184 | 3,512 | 66 | 62 | 57 | 68 | 80 |
| Rio Grande do Sul | 6,447 | 6,605 | 6,870 | 7,368 | 8,136 | 328 | 270 | 224 | 195 | 264 |
| Santa Catarina | 2,046 | 1,982 | 2,012 | 2,508 | 2,273 | 89 | 89 | 99 | 94 | 101 |
| **Subtotal** | **11,359** | **11,510** | **11,890** | **13,060** | **13,921** | **483** | **421** | **380** | **357** | **445** |
| **Northeast Region** | | | | | | | | | | |
| Alagoas | 596 | 632 | 695 | 748 | 793 | 10 | 16 | 20 | 15 | 11 |
| Bahia | 3,748 | 3,845 | 3,952 | 4,578 | 5,028 | 158 | 148 | 128 | 136 | 165 |
| Ceará | 2,284 | 2,203 | 2,186 | 2,032 | 2,359 | 37 | 45 | 44 | 45 | 61 |
| Maranhão | 935 | 1,079 | 1,224 | 1,276 | 1,408 | 13 | 15 | 14 | 19 | 28 |
| Paraíba | 1,597 | 1,676 | 1,802 | 2,131 | 2,244 | 40 | 40 | 32 | 28 | 36 |
| Pernambuco | 2,472 | 2,600 | 2,688 | 2,720 | 3,054 | 39 | 32 | 22 | 23 | 25 |
| Piauí | 618 | 691 | 762 | 844 | 983 | 11 | 6 | 4 | 9 | 15 |
| Rio Grande do Norte | 1,476 | 1,547 | 1,593 | 1,741 | 1,986 | 94 | 94 | 97 | 97 | 117 |
| Sergipe | 396 | 418 | 448 | 492 | 577 | 4 | 5 | 5 | 5 | 6 |
| **Subtotal** | **14,122** | **14,691** | **15,350** | **16,562** | **18,432** | **406** | **401** | **366** | **377** | **464** |
| **Center-West Region** | | | | | | | | | | |
| Federal District | 917 | 949 | 904 | 834 | 1,135 | 34 | 33 | 18 | 21 | 11 |
| Goiás | 2,875 | 3,153 | 3,544 | 3,990 | 4,281 | 187 | 173 | 107 | 93 | 107 |
| Mato Grosso | 419 | 444 | 453 | 476 | 604 | 72 | 76 | 49 | 50 | 30 |
| Mato Grosso do Sul | 350 | 369 | 359 | 1,154 | 550 | 30 | 27 | 21 | 20 | 26 |
| **Subtotal** | **4,561** | **4,915** | **5,260** | **6,454** | **6,570** | **323** | **309** | **195** | **184** | **174** |
| **North Region** | | | | | | | | | | |
| Acre | 1 | 1 | 1 | 1 | 4 | 0 | 0 | 0 | 0 | 0 |
| Amazonas | 49 | 39 | 29 | 27 | 32 | 4 | 3 | 1 | 1 | 1 |
| Amapá | 17 | 20 | 23 | 25 | 51 | 0 | 0 | 1 | 1 | 1 |
| Pará | 1,224 | 1,365 | 1,538 | 1,529 | 1,863 | 21 | 22 | 47 | 166 | 135 |
| Rondônia | 122 | 149 | 199 | 263 | 208 | 2 | 1 | 2 | 12 | 22 |
| Roraima | 15 | 23 | 18 | 18 | 32 | 1 | 0 | 0 | 0 | 1 |
| Tocantins | 59 | 70 | 91 | 112 | 151 | 3 | 4 | 4 | 4 | 6 |
| **Subtotal** | **1,487** | **1,667** | **1,899** | **1,975** | **2,341** | **31** | **30** | **55** | **184** | **166** |
| **Total (Brazil)** | **58,424** | **61,434** | **64,328** | **69,173** | **73,576** | **1,850** | **1,770** | **1,516** | **1,606** | **1,612** |

**Abbreviations:** eL/B, Patient-equivalent estimates of people receiving L/B in the reference year; eL/C, Patient-equivalent estimates of people receiving L/C in the reference year. The estimated values between 0 and 1 were rounded to 1.

**B. Sensitivity analyses after -10% in daily tablet use: patient-equivalent estimates of people receiving L/B (5.24 tablets/day) and L/C (4.07 tablets/day) from 2020 to 2024:**

| **State** | **eL/B 2020** | **eL/B 2021** | **eL/B 2022** | **eL/B 2023** | **eL/B 2024** | **eL/C 2020** | **eL/C 2021** | **eL/C 2022** | **eL/C 2023** | **eL/C 2024** |
| --- | --- | --- | --- | --- | --- | --- | --- | --- | --- | --- |
| **Southeast Region** | | | | | | | | | | |
| Espírito Santo | 1,127 | 1,237 | 1,274 | 1,386 | 1,311 | 24 | 48 | 90 | 168 | 30 |
| Minas Gerais | 6,933 | 7,244 | 7,734 | 7,881 | 8,013 | 207 | 208 | 164 | 146 | 148 |
| Rio de Janeiro | 9,291 | 10,146 | 10,209 | 10,839 | 11,184 | 161 | 149 | 94 | 78 | 61 |
| São Paulo | 12,519 | 13,199 | 14,021 | 14,460 | 15,378 | 270 | 263 | 216 | 159 | 152 |
| **Subtotal** | **29,870** | **31,826** | **33,239** | **34,566** | **35,887** | **662** | **668** | **564** | **550** | **391** |
| **South Region** | | | | | | | | | | |
| Paraná | 3,184 | 3,248 | 3,342 | 3,538 | 3,902 | 73 | 69 | 63 | 76 | 89 |
| Rio Grande do Sul | 7,163 | 7,339 | 7,633 | 8,187 | 9,040 | 364 | 300 | 249 | 217 | 293 |
| Santa Catarina | 2,273 | 2,202 | 2,236 | 2,787 | 2,526 | 99 | 99 | 110 | 104 | 112 |
| **Subtotal** | **12,621** | **12,789** | **13,211** | **14,511** | **15,468** | **537** | **468** | **422** | **397** | **494** |
| **Northeast Region** | | | | | | | | | | |
| Alagoas | 662 | 702 | 772 | 831 | 881 | 11 | 18 | 22 | 17 | 12 |
| Bahia | 4,164 | 4,272 | 4,391 | 5,087 | 5,587 | 176 | 164 | 142 | 151 | 183 |
| Ceará | 2,538 | 2,448 | 2,429 | 2,258 | 2,621 | 41 | 50 | 49 | 50 | 68 |
| Maranhão | 1,039 | 1,199 | 1,360 | 1,418 | 1,564 | 14 | 17 | 16 | 21 | 31 |
| Paraíba | 1,774 | 1,862 | 2,002 | 2,368 | 2,493 | 44 | 44 | 36 | 31 | 40 |
| Pernambuco | 2,747 | 2,889 | 2,987 | 3,022 | 3,393 | 43 | 36 | 24 | 26 | 28 |
| Piauí | 687 | 768 | 847 | 938 | 1,092 | 12 | 7 | 4 | 10 | 17 |
| Rio Grande do Norte | 1,640 | 1,719 | 1,770 | 1,934 | 2,207 | 104 | 104 | 108 | 108 | 130 |
| Sergipe | 440 | 464 | 498 | 547 | 641 | 4 | 6 | 6 | 6 | 7 |
| **Subtotal** | **15,691** | **16,323** | **17,056** | **18,402** | **20,480** | **451** | **446** | **407** | **419** | **516** |
| **Center-West Region** | | | | | | | | | | |
| Federal District | 1,019 | 1,054 | 1,004 | 927 | 1,261 | 38 | 37 | 20 | 23 | 12 |
| Goiás | 3,194 | 3,503 | 3,938 | 4,433 | 4,757 | 208 | 192 | 119 | 103 | 119 |
| Mato Grosso | 466 | 493 | 503 | 529 | 671 | 80 | 84 | 54 | 56 | 33 |
| Mato Grosso do Sul | 389 | 410 | 399 | 1,282 | 611 | 33 | 30 | 23 | 22 | 29 |
| **Subtotal** | **5,068** | **5,461** | **5,844** | **7,171** | **7,300** | **359** | **343** | **217** | **204** | **193** |
| **North Region** | | | | | | | | | | |
| Acre | 1 | 1 | 1 | 1 | 4 | 0 | 0 | 0 | 0 | 0 |
| Amazonas | 54 | 43 | 32 | 30 | 36 | 4 | 3 | 1 | 1 | 1 |
| Amapá | 19 | 22 | 26 | 28 | 57 | 0 | 0 | 1 | 1 | 1 |
| Pará | 1,360 | 1,517 | 1,709 | 1,699 | 2,070 | 23 | 24 | 52 | 184 | 150 |
| Rondônia | 136 | 166 | 221 | 292 | 231 | 2 | 1 | 2 | 13 | 24 |
| Roraima | 17 | 26 | 20 | 20 | 36 | 1 | 0 | 0 | 0 | 1 |
| Tocantins | 66 | 78 | 101 | 124 | 168 | 3 | 4 | 4 | 4 | 7 |
| **Subtotal** | **1,652** | **1,852** | **2,110** | **2,194** | **2,601** | **34** | **33** | **61** | **204** | **184** |
| **Total (Brazil)** | **64,916** | **68,260** | **71,476** | **76,859** | **81,751** | **2,056** | **1,967** | **1,684** | **1,784** | **1,791** |

**Abbreviations:** eL/B, Patient-equivalent estimates of people receiving L/B in the reference year; eL/C, Patient-equivalent estimates of people receiving L/C in the reference year. The estimated values between 0 and 1 were rounded to 1.

**C. Sensitivity analyses after +10% in daily tablet use: patient-equivalent estimates of people receiving L/B (6.41 tablets/day) and L/C (4.98 tablets/day) from 2020 to 2024:**

| **State** | **eL/B 2020** | **eL/B 2021** | **eL/B 2022** | **eL/B 2023** | **eL/B 2024** | **eL/C 2020** | **eL/C 2021** | **eL/C 2022** | **eL/C 2023** | **eL/C 2024** |
| --- | --- | --- | --- | --- | --- | --- | --- | --- | --- | --- |
| **Southeast Region** | | | | | | | | | | |
| Espírito Santo | 922 | 1,012 | 1,043 | 1,134 | 1,073 | 20 | 39 | 74 | 137 | 25 |
| Minas Gerais | 5,673 | 5,927 | 6,328 | 6,448 | 6,556 | 169 | 170 | 135 | 119 | 121 |
| Rio de Janeiro | 7,602 | 8,301 | 8,353 | 8,868 | 9,151 | 132 | 122 | 77 | 64 | 50 |
| São Paulo | 10,243 | 10,799 | 11,472 | 11,831 | 12,582 | 221 | 215 | 176 | 130 | 125 |
| **Subtotal** | **24,439** | **26,039** | **27,195** | **28,281** | **29,362** | **542** | **546** | **462** | **450** | **320** |
| **South Region** | | | | | | | | | | |
| Paraná | 2,605 | 2,657 | 2,735 | 2,895 | 3,193 | 60 | 56 | 52 | 62 | 73 |
| Rio Grande do Sul | 5,861 | 6,005 | 6,245 | 6,698 | 7,396 | 298 | 245 | 204 | 177 | 240 |
| Santa Catarina | 1,860 | 1,802 | 1,829 | 2,280 | 2,066 | 81 | 81 | 90 | 85 | 92 |
| **Subtotal** | **10,326** | **10,464** | **10,809** | **11,873** | **12,655** | **439** | **383** | **345** | **325** | **405** |
| **Northeast Region** | | | | | | | | | | |
| Alagoas | 542 | 575 | 632 | 680 | 721 | 9 | 15 | 18 | 14 | 10 |
| Bahia | 3,407 | 3,495 | 3,593 | 4,162 | 4,571 | 144 | 135 | 116 | 124 | 150 |
| Ceará | 2,076 | 2,003 | 1,987 | 1,847 | 2,145 | 34 | 41 | 40 | 41 | 55 |
| Maranhão | 850 | 981 | 1,113 | 1,160 | 1,280 | 12 | 14 | 13 | 17 | 25 |
| Paraíba | 1,452 | 1,524 | 1,638 | 1,937 | 2,040 | 36 | 36 | 29 | 25 | 33 |
| Pernambuco | 2,247 | 2,364 | 2,444 | 2,473 | 2,776 | 35 | 29 | 20 | 21 | 23 |
| Piauí | 562 | 628 | 693 | 767 | 894 | 10 | 5 | 4 | 8 | 14 |
| Rio Grande do Norte | 1,342 | 1,406 | 1,448 | 1,583 | 1,805 | 85 | 85 | 88 | 88 | 106 |
| Sergipe | 360 | 380 | 407 | 447 | 525 | 4 | 5 | 5 | 5 | 5 |
| **Subtotal** | **12,838** | **13,355** | **13,955** | **15,056** | **16,756** | **369** | **365** | **333** | **343** | **422** |
| **Center-West Region** | | | | | | | | | | |
| Federal District | 834 | 863 | 822 | 758 | 1032 | 31 | 30 | 16 | 19 | 10 |
| Goiás | 2,614 | 2,866 | 3,222 | 3,627 | 3,892 | 170 | 157 | 97 | 85 | 97 |
| Mato Grosso | 381 | 404 | 412 | 433 | 549 | 65 | 69 | 45 | 45 | 27 |
| Mato Grosso do Sul | 318 | 335 | 326 | 1,049 | 500 | 27 | 25 | 19 | 18 | 24 |
| **Subtotal** | **4,146** | **4,468** | **4,782** | **5,867** | **5,973** | **294** | **281** | **177** | **167** | **158** |
| **North Region** | | | | | | | | | | |
| Acre | 1 | 1 | 1 | 1 | 4 | 0 | 0 | 0 | 0 | 0 |
| Amazonas | 45 | 35 | 26 | 25 | 29 | 4 | 3 | 1 | 1 | 1 |
| Amapá | 15 | 18 | 21 | 23 | 46 | 0 | 0 | 1 | 1 | 1 |
| Pará | 1,113 | 1,241 | 1,398 | 1,390 | 1,694 | 19 | 20 | 43 | 151 | 123 |
| Rondônia | 111 | 135 | 181 | 239 | 189 | 2 | 1 | 2 | 11 | 20 |
| Roraima | 14 | 21 | 16 | 16 | 29 | 1 | 0 | 0 | 0 | 1 |
| Tocantins | 54 | 64 | 83 | 102 | 137 | 3 | 4 | 4 | 4 | 5 |
| **Subtotal** | **1,352** | **1,515** | **1,726** | **1,795** | **2,128** | **28** | **27** | **50** | **167** | **151** |
| **Total (Brazil)** | **53,113** | **55,849** | **58,480** | **62,885** | **66,887** | **1,682** | **1,609** | **1,378** | **1,460** | **1,465** |

**Abbreviations:** eL/B, Patient-equivalent estimates of people receiving L/B in the reference year; eL/C, Patient-equivalent estimates of people receiving L/C in the reference year. The estimated values between 0 and 1 were rounded to 1.
